# Enhancing the Detection of Dysmorphic Red Blood Cells and Renal Tubular Epithelial Cells with a Modified Urinalysis Protocol

**DOI:** 10.1038/srep40521

**Published:** 2017-01-11

**Authors:** Yu Chu-Su, Kenichi Shukuya, Takashi Yokoyama, Wei-Chou Lin, Chih-Kang Chiang, Chii-Wann Lin

**Affiliations:** 1 Institute of Biomedical Engineering, National Taiwan University, No. 1, Sec. 4, Roosevelt Rd., Taipei City 10617, Taiwan; 2 Department of Laboratory Medicine, National Taiwan University Hospital, No. 7, Zhongshan S. Rd., Taipei City 10002, Taiwan; 3 Department of Clinical Laboratory, The University of Tokyo Hospital, 7-3-1, Hongo, Bunkyo-ku, Tokyo 113-8655, Japan; 4 Department of Central Clinical Laboratory, Tokyo Women’s Medical University Hospital, 8-1, Kawada-cho, Shinjyuku-ku, Tokyo 162-8666, Japan; 5 Department of Pathology and Graduate Institute of Pathology, College of Medicine, National Taiwan University, No. 7, Zhongshan S. Rd., Taipei City 10002, Taiwan; 6 Graduate Institute of Toxicology, College of Medicine, No. 1, Jen-Ai Rd., Taipei City 10002, Taiwan; 7 Department of Integrated Diagnostics & Therapeutics, National Taiwan University Hospital, No. 7, Zhongshan S. Rd., Taipei City 10002, Taiwan

## Abstract

Urinary sediment is used to evaluate patients with possible urinary tract diseases. Currently, numerous protocols are applied to detect dysmorphic red blood cells (RBCs) and renal tubular epithelial cells (RTECs) in urinary sediment. However, distinct protocols are used by nephrologists and medical technologists for specimen concentration and observation, which leads to major discrepancies in the differential counts of formed elements such as dysmorphic RBCs and RTECs and might interfere with an accurate clinical diagnosis. To resolve these problems, we first tested a modified urinalysis protocol with an increased relative centrifuge force and concentration factor in 20 biopsy-confirmed glomerulonephritis patients with haematuria. We successfully improved the recovery ratio of dysmorphic RBCs in clinical specimens from 34.7% to 42.0% (P < 0.001). Furthermore, we confirmed the correlation between counts by the modified urinary protocol and Sysmex UF-1000i urinary flow cytometer (r ≥ 0.898, P < 0.001). A total of 28 types of isomorphic and dysmorphic RBCs were detected using a bright field microscope, with results comparable to those using a standard phase contrast microscope. Finally, we applied Sternheimer stain to enhance the contrast of RTECs in the urinary sediments. We concluded that this modified urinalysis protocol significantly enhanced the quality of urinalysis.

Urinalysis is traditionally performed in a central laboratory by certified medical laboratory technologists using an automatic urinalysis system^1^. Urinary sediment microscopy plays a significant role in the clinical diagnosis of urinary tract diseases^2^,^3^,^4^. Several standardized protocols for urinary sediment microscopy have been proposed by different professional societies^5^,^6^,^7^,^8^,^9^.

In 2005, nephrologists began reporting concerns about the frequent discrepancies in reported results of urine sediment microscopy^1^,^10^. For instance, the rates of correct diagnosis of acute kidney injury (AKI) based on reports by medical technologists and nephrologists were <25% and 69–92%, respectively^1^. These differences could be caused by the number of renal tubular epithelial (RTE) cells identified, RTE cell casts, and granular casts, all of which tend to be underestimated by laboratory technologists. In addition, RTE cells are often misclassified as squamous epithelial cells, and, usually, the reports issued by medical laboratory technologists are missing a description of dysmorphic red blood cells (RBCs).

Nephrologists may prioritize results of urinary sediment microscopy for diagnosis, and they will apply protocols to obtain highly concentrated urinary sediments, which may result in lower reproducibility due to volumetric variation^11^. In contrast, technologists in medical laboratories may be concerned primarily with accreditation by the College of American Pathologists (CAP), and therefore with proficiency tests for the accuracy of RBC and white blood cell (WBC) counts. Thus, medical laboratory technologists adhere to protocols issued by organizations related to laboratory medicine, which may result in underestimating the formed elements in urine sediment. Some researchers report that concentrating urine sediment can increase the rate of dysmorphic RBCs found in a specimen^12^. However, differences in specimen preparation procedures before microscopic examination may be an important cause of such discrepancies between the reports by nephrologists and medical technologists. Thus, these discrepancies should be investigated to standardize these protocols^13^.

In 1979, Fairley and Birch first depicted the morphological characteristics of dysmorphic RBCs resulting from glomerular bleeding^14^,^15^. The prominent features of dysmorphic RBCs are variously shaped blebs or projections at the cell membrane. Some researchers classify these dysmorphic RBCs as acanthocytes, G1 cells, or D cells based on the characteristic shapes^15^,^16^,^17^,^18^. In contrast, the morphological features of isomorphic RBCs resulting from renal pelvis, ureter, or bladder bleeding are uniform and no more than two types^17^,^19^. Thus, different medical laboratories citing different references can lead to differences in the ability to identify dysmorphic RBCs, which could be a reason that reports by medical laboratory technologists lack a description of dysmorphic RBCs. Therefore, the shapes of dysmorphic and isomorphic RBCs should be systemically investigated.

In addition, according to the reference published by CAP for urinary sediment microscopy, medical laboratory technologists do not routinely apply a stain to enhance the contrast between different formed elements in urinary sediment^20^.

Based on our clinical observations, we proposed a modified protocol to increase the accuracy of identifying dysmorphic RBCs and discriminating RTE cells from squamous epithelial cells. This modified protocol was validated using clinical samples and is expected to enhance the reporting quality of urinary sediment microscopy.

## Methods

This study was approved by the Institutional Review Board of National Taiwan University Hospital (200708070 R). All volunteers (n = 280) provided written informed consent before enrolling in this study. All of the enrolees were more than 20 years old, free of malignancies, confirmed to have type 2 diabetes mellitus, and were regularly followed for more than three months between June 2010 and January 2011. Among these participants, 40 were enrolled because of haematuria from August to November 2010^21^. Twenty were diagnosed with glomerulonephritis on renal biopsy, and the other 20 were diagnosed with urinary tract infection according to the results of urine culture or calculi in the urinary tract observed by ultrasonography^22^. Another 240 patients were enrolled between December 2010 and January 2011. All participants were informed that 50 mL of random mid-stream urine samples would be collected using a 50-mL sterile capped container for analysis by urine sediment microscopy.

The results of urinary sediment microscopy were affected by the urinary sediment preparation procedures, including the sample volume, relative centrifuge force (RCF), centrifuge duration, concentration factor, and method of observation. Nephrologists apply high concentration protocols for urine sediment preparation. For example, 10 mL urine was centrifuged at 1,500–3,000 revolutions per minute (RPM) for 3–10 min, and the pellet was resuspended with a minimal volume of supernatant (0.3–0.5 mL). Then, a drop of the suspension was placed on a glass slide with a cover slip for observation^1^,^5^,^23^,^24^.

In contrast, medical laboratory technologists apply a protocol recommended by Clinical and Laboratory Standards Institute (CLSI), GP-16A3^7^. Under this protocol, 12 mL of urine was centrifuged at an RCF of 400× *g* for 5 min, and the pellet was resuspended with 1 mL of supernatant. Therefore, the concentration factor was only 12-fold, which was lower than that in the nephrologists’ general protocol. Then, a drop of suspension was loaded into a disposable chamber slide for observation^1^,^7^.

We surveyed three published guidelines (the European Urinalysis Guidelines [EUG] by the European Confederation of Laboratory Medicine^8^, Urinalysis Approved Guideline GP16-A3 by CLSI^7^, and Urine Sediment Microscopy Guideline GP1-P4 by the Japanese Committee for Clinical Laboratory Standards^9^), and we compared these guidelines with nephrologist’s methods to propose a modified protocol (or standard operating procedure) for urinary sediment microscopy^1^,^5^,^15^,^17^,^23^. This modified method of urinary sediment microscopy in the study was carried out in accordance with these guidelines (EUG, GP16-A3, and GP1-P4), which was approved by the Institutional Review Board of National Taiwan University Hospital (200708070 R).

Most of the cited guidelines suggest that 10 mL of urine is optimal for urine sediment microscopy. Regarding the RCF used, there are discrepancies between the methods used by nephrologists and medical technologists. Nephrologists define RCF by RPM. Because the RCF varies with the radius of the rotor, the RCF is defined by relative gravity, *g*, by medical technologists. The conversion formula between RCF (*g*) and RPM is as follows: RCF(*g*) = 1.118 × 10^−5^ × radius (cm) × RPM^2^,^7^,^8^,^9^. In the three guidelines, two settings are suggested for RCF, 400× *g* and 500× *g*, and we compared the performance of the methods using these two RCF settings to identify the optimal centrifugation speed. All three guidelines recommend centrifugation for 5 min. Because the concentration factors for urinary formed elements and the methods of sample loading for microscopy affect the counting results, the concentration factor and sample loading method were also optimized, based on the current guidelines. As a highly concentrated specimen will increase the potential to detect RTEs in a high-power field (HPF), a 20-fold concentration factor is recommended by nephrologists and the EUG as a suitable concentration factor^8^,^17^,^23^.

For sample loading, some nephrologists prefer to apply a drop of urinary sediment suspension under the cover slip of glass slides^1^,^23^,^25^. Because the volume of the drop is not well defined, variations in the height between the cover slip and glass slide can lead to differences in the examined volume, which compromises the reproducibility of the count^13^. Two guidelines (EUG and GP16 A-3) suggest using a haemocytometer or a disposable slide with 10 counting chambers for microscopic observation^7^,^8^,^15^,^17^. Two guidelines (EUG and GP1-P4) also suggest using Sternheimer stain to increase the contrast between different formed elements in urinary sediment^8^,^9^,^26^.

Various shapes of dysmorphic RBCs had been recorded and identified, but there is no consensus standard^15^,^16^,^17^,^25^. In addition, there is no definition or reference images of dysmorphic RBCs, and dysmorphic RBCs are not a recommended item for reporting by GP-16A3. To the best of our knowledge, GP1-P4 was the first guideline to describe the shapes of dysmorphic RBCs in urine sediment^9^. We used the shapes of isomorphic and dysmorphic RBCs described in the GP1-P4 guideline as a reference to identify dysmorphic RBCs.

Forty urine samples from 40 volunteers with haematuria were used to identify a suitable RCF^21^. Fifty millilitres of collected urine was mixed well and transferred in equal volumes to five commercialized urine centrifuge tubes (Shin-Yung Medical Instrument Co., Taiwan). One un-centrifuged aliquot was analysed using a Sysmex UF-1000i urine flow cytometer (Sysmex Co., Tokyo, Japan) to detect formed elements in urine. Two aliquots were centrifuged (E4000, Kubota, Co., Tokyo, Japan) at 400× *g* (1,450 RPM), and two aliquots were centrifuged at 500× *g* (1,620 RPM). After centrifugation, the paired supernatants were decanted into two new 50-mL sterile capped containers. The two tubes of well-mixed supernatants were analysed using the Sysmex UF-1000i urine flow cytometer.

After 9.5 mL of supernatant was decanted from the commercialized urine centrifuge tube, 0.5 mL of supernatant with a pellet was trapped in the bottom of the tube with a designed capillary valve so that the sample was concentrated 20-fold. Then, the pellet was resuspended in the supernatant. Paired suspensions were transferred into two new 1.5-mL sterile Eppendorf tubes and analysed using the Sysmex UF-1000i urine flow cytometer. The residual and recovery ratios of the urinary formed elements (RBCs, WBCs, epithelial cells [ECs]) in the supernatant and suspension were compared to determine a suitable RCF.

When the RCF was chosen, the formed elements in 80 urine samples were counted, and correlations between the results of manual observation with a disposable chamber slide (Shin-Yung Medical Instrument Co., Taiwan) and those of the Sysmex UF-1000i urine flow cytometer were determined. Parameters for a count per microliter to a count per HPF were determined.

When the conversion factor from microliter to HPF was set for the Sysmex UF-1000i urine flow cytometer, 160 urine samples were tested, and the results were pooled with those of the previous 80 samples to determine the agreement between results by manual observation with a disposable chamber slide and those using the Sysmex UF-1000i urine flow cytometer and CLSI GP 16-A3 guideline.

In addition, the shapes of RBCs were observed under bright field and phase contrast microscopes, and results were recorded. Ninety microliters of residual urine sediment suspension from each sample was transferred into a new Eppendorf tube and mixed with 10 μL of Sternheimer stain (no. 5018–2, Muto Pure Chemical Co., Tokyo, Japan) to evaluate cellular morphology. All data were analysed using SigmaPlot 12 (Systat Software Inc., San Jose, CA).

## Results

### Centrifuge force affects the recovery rate of dysmorphic RBCs

Further classification of RBCs into dysmorphic RBCs and isomorphic RBCs according to the calculated residual ratio and recovery ratio are shown in Table 1.

The residual ratios of dysmorphic and isomorphic RBCs in the supernatants from patients with haematuria at an RCF of 500× *g* decreased significantly from the values obtained at an RCF of 400× *g* (from 30.8% to 24.1% [*P* = 0.002] and from 10.7% to 5.1% [*P* = 0.001], respectively). Furthermore, an RCF of 500× *g* significantly increased the sediment recovery ratios of dysmorphic RBCs and isomorphic RBCs from the values observed at an RCF of 400× *g* (from 34.7% to 42.0% [*P* < 0.001] and from 53.7% to 57.7% [*P* = 0.002], respectively). Based on this evidence, an RCF of 500× *g* is preferable.

### Results of the modified protocol for manual urinary sediment microscopy show good agreement with those from the Sysmex UF-1000i urine flow cytometer

**Figure 1:**
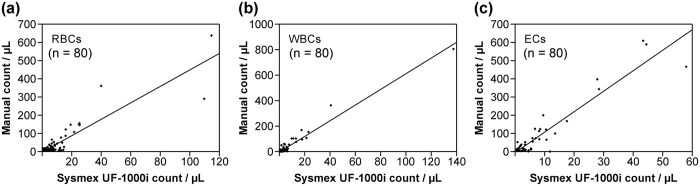
Count correlations between the Sysmex UF-1000i (μL) and manual observation with disposable chamber slide (μL) for red blood cells (RBCs), white blood cells (WBCs), and epithelial cells (ECs). (**a**), The equation that unified manual (μL) and Sysmex UF-1000i (μL) RBC counts was the following: Manual count = −5.468 + (5.049 × Sysmex UF-1000i count). The r value was 0.898 (P < 0.001), and the r^2^ value was 0.807. (**b**), The equation that unified manual (μL) and Sysmex UF-1000i (μL) WBC counts was the following: Manual count = 0.667 + (6.825 × Sysmex UF-1000i count). The r value was 0.977 (P < 0.001), and the r^2^ value was 0.955. (**c**), The equation that unified manual (μL) and Sysmex UF-1000i (μL) RBC counts was the following: Manual count = −7.450 + (12.559 × Sysmex UF-1000i count). The r value was 0.947 (P < 0.001); and the r^2^ value was 0.898.

Count correlations for RBCs, WBCs, and ECs by manual observation with the disposable chamber slide per microliter (μL) and using the Sysmex UF-1000i urine flow cytometer (μL) are shown in Fig. 1. The r values of the RBC (Fig. 1a), WBC (Fig. 1b), and EC (Fig. 1c) counts by manual observation and the Sysmex UF-1000i urine flow cytometer were 0.898, 0.977, and 0.947, respectively, and the r^2^ values of the counts of RBCs, WBCs, and ECs were 0.807, 0.955, and 0.898, respectively.

The r values of counts for RBCs, WBCs, and ECs by manual observation and Sysmex UF-1000i urine flow cytometer were all over 0.898 with *P* < 0.001; thus, the count correlation between manual observation and Sysmex UF-1000i urine flow cytometry analysis using this modified protocol is acceptable. In addition, the parameter settings for the Sysmex UF-1000i urine flow cytometer used to convert a count value per microliter to a count per HPF were optimized.

The agreement in counts of 240 samples by manual observation with disposable chamber slide (HPF) and using the Sysmex UF-1000i urine flow cytometer (HPF) is shown in Tables 2, 3 and 4. Regarding the RBC count (Table 2), 90.4% of sample results were in complete agreement using the two methods, 99.2% of sample results were within ±1 agreement, and only two cases were outside of this range. Regarding the WBC count (Table 3), 94.6% of sample results were in complete agreement using the two methods, 99.2% of sample results were within ±1 agreement, and only two cases were outside of this range. Regarding the EC count (Table 4), 92.1% of sample results were in complete agreement using the two methods, and 100% of sample results were within ±1 agreement. Agreement within the ±1 reporting range for all three cells types was over 99%; thus, the cell counts based on the modified protocol for urine sediment microscopy are reliable.

### Twenty-eight types of isomorphic and dysmorphic RBCs were detected by bright field microscope

**Figure 2:**
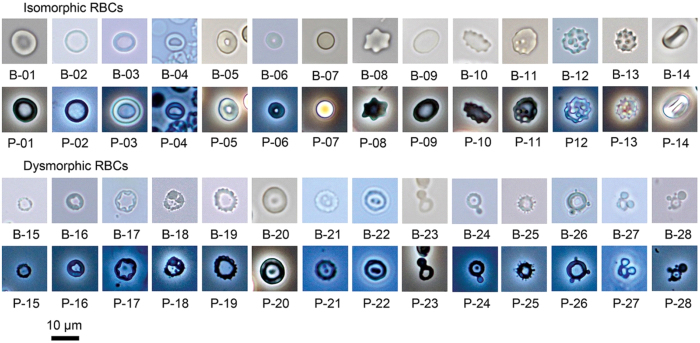
Isomorphic red blood cells (RBCs) and dysmorphic RBCs, 400×. B: bright field microscope; P: phase contrast microscope. Images 01–14 are isomorphic RBCs, and images 15–28 are dysmorphic RBCs.

Images of isomorphic RBCs and dysmorphic RBCs are shown in Fig. 2. All RBCs were assessed using bright field and phase contrast microscopes. The standard shape of an isomorphic RBC was a bi-concave disk (Fig. 2B-01), and the diameter of an isomorphic RBCs’ central pale was about half of the diameter of an RBC.

The shapes of isomorphic RBCs can change in response to the osmolarity of urine. The isomorphic RBCs swell to spheres (Fig. 2B-07) in urine with a low specific gravity, and they shrink to the shape of a spiked disk (Fig. 2B-11 and B-12) or a spiked sphere (Fig. 2B-13) in urine with a high specific gravity. When the central pale is present, its margin is smooth in isomorphic RBCs (Fig. 2B-01 and B-06). In contrast, the margin of the central pale is uneven (Fig. 2B-15 to B-18) or the central pale has a spot like a target (Fig. 2B-19 to B-22) in dysmorphic RBCs. In addition, some dysmorphic RBCs have bleb projections (Fig. 2B-23 to B-28) at the margin. As shown in Fig. 2, all of the images of dysmorphic RBCs from bright field microscope were as clear as those from the phase contrast microscope.

### Sternheimer stain enhances the contrast between different formed elements in urinary sediment

Sternheimer stain is a supervital stain, and the major components are national fast blue (Alcian blue) and Pyronin B solutions. Alcian blue causes the nucleus or the matrix of a hyaline cast to turn blue, whereas Pyronin B causes the cytoplasm or granules in a granular cast to turn pink-red or dense purple. Because the cytoplasmic membrane of living cells has selective permeability, a living cell cannot be stained. When the cell is damaged, the cytoplasm will stain pale pink. If the cell is dead, the nucleus becomes blue, and the cytoplasm will stain pale pink to dense purple-blue, depending on the type of cell.

**Figure 3:**
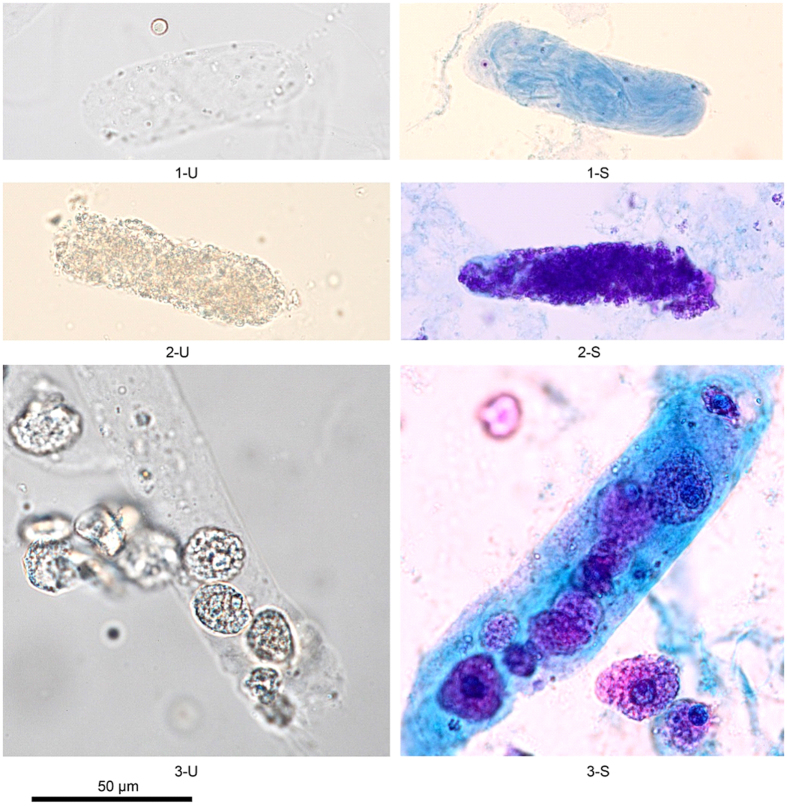
Casts, 400×. U, unstained; S, Sternheimer stain. (1-U) Hyaline cast: the matrix of the hyaline cast is transparent with two rounded ends. (1-S) After staining, the contrast increases, and the matrix of the hyaline cast is light blue. (2-U) Granular cast: the colourless matrix has many granules. (2-S) After staining, the contrast increases, the matrix of the cast is light blue, and the granules are dense purple. (3-U) Renal tubular epithelial (RTE) cell cast: the colourless matrix of the cast has four renal tubular epithelial cells. (3-S) After staining, the contrast increases, and the matrix of the RTE cell cast is light blue. The cytoplasm of an RTE cell is pink-red, and the nucleus of an RTE cell is cyan blue.

**Figure 4:**
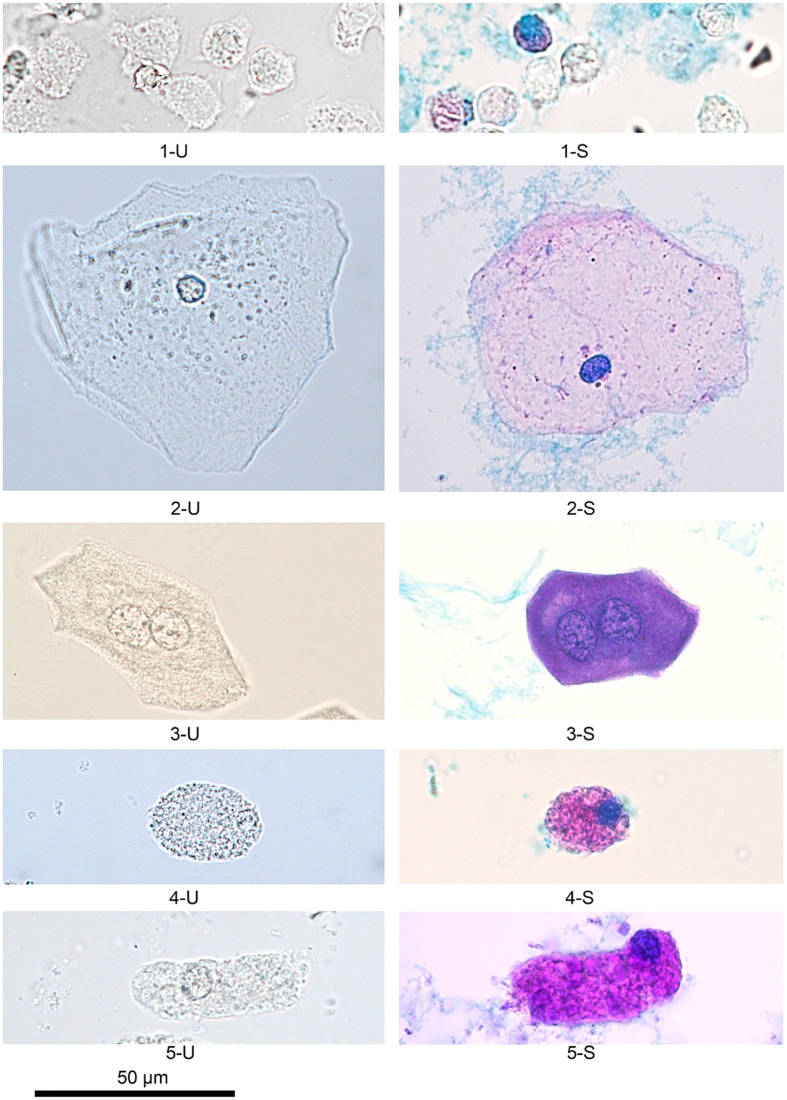
Various cells, 400×. U, unstained; S, Sternheimer stain. (1-U) Active neutrophils vary in shape from amoeboid to round. (1-S) After staining, active neutrophils are round. The cytoplasm and nucleus are colourless. When the cells are damaged, the cytoplasm will be pale pink, and the nucleus will be dense blue. (2-U) Squamous epithelial cell: the size of its nucleus is about that of a red blood cell. The cellular margin is polygonal, and the cytoplasmic clarity is high, with a homogenous surface. (2-S) After staining, the cytoplasm is pale pink, and the nucleus is dense blue. (3-U) Urothelial cell: the nucleus is 1.5 times that of a white blood cell, and the nucleolus is prominent. The cytoplasmic clarity is low. The cellular margin is distinctly shaped, curved, and polygonal. (3-S) After staining, the cytoplasm is dense purple, and the nucleus is purple-blue. (4-U and 5-U) Renal tubular epithelial cell: The nucleus is shrunken and eccentric, and the cytoplasm is sandy with granules. The cellular margin is uneven. (4-S and 5-S) After staining, the cytoplasm is pink-red, and the nucleus is cyan blue and very eccentric.

Results of Sternheimer staining of the cells and casts are shown in Figs 3 and 4. Characteristics of the stained cells are shown in Table 5. In general, the Sternheimer stain enhanced the contrast of cells and casts. After staining, the characteristics of different cells and casts were easily distinguished, increasing the potential to identify various formed elements in urine sediment.

## Discussion

In general, nephrologists apply a high centrifuge force and concentration factor in analysing urinary sediments to increase the potential to find important elements such as dysmorphic RBCs and RTE cells^1^,^15^,^27^. In contrast, medical laboratory technologists follow the CLSI-recommended protocol, which specifies a lower centrifuge force and concentration factor. This recommended protocol decreases the potential to find dysmorphic RBCs and RTE cells. In practice, urinary sediment microscopy is usually performed by junior technologists in medical laboratories^19^. Without proper reference images of the shapes of dysmorphic RBCs or a staining technique to increase the contrast between different formed elements in urinary sediment, junior medical laboratory technologists can underestimate the counts of dysmorphic RBCs and RTE cells. In addition, a precise count of dysmorphic RBCs is very important for evaluating glomerular bleeding before renal biopsy^19^.

Our proposed modified urinalysis protocol increases the detection rate of dysmorphic red blood cells and RTE cells. The following points indicate the value of the modified protocol. First, the recovery ratios from sediment suspension and the residual ratios in the supernatant of formed elements in urine were evaluated and they are shown in Supplementary Table 1. For RBCs, WBCs, and ECs, the sum of the recovery ratio and residual ratio for three cells of each type was in a range from 63.1% to 71.6%. This implies that cells of each type were degraded during centrifugation at both of the RCF settings. Therefore, an RCF with higher recovery ratios for formed elements in urine sediment is preferable to increase the cell count accuracy. The recovery ratio of RBCs from the sediment suspension at an RCF of 500× *g* (49.8%) was significantly higher (*P* < 0.001) than that at an RCF of 400× *g* (44.2%), whereas their residual ratio at an RCF of 500× *g* (14.6%) was significantly lower (*P* < 0.001) than that at an RCF of 400× *g* (20.8%). Even so, the recovery ratio of RBCs was the lowest of the three urinary formed elements (RBC, WBCs, and ECs) in the sediment suspension. There is a loss of haemoglobin in dysmorphic RBCs, which results in a specific gravity of dysmorphic RBCs that is lower than that of isomorphic RBCs^19^. Thus, the recovery ratio of RBCs was the lowest, which caused the RBC count correlation drop to 0.807 (WBC = 0.955 and EC = 0.898). In addition, the RCF of 500× *g* significantly increased the recovery ratio of WBCs in the sediment suspension (*P* = 0.026) and significantly decreased the residual ratio of WBCs in the supernatant (*P* = 0.032). However, there was no significant difference between the recovery and residual ratios of ECs using the different RCF settings, which could be that the weight of EC is heavier than RBC and WBC thus the RCF of 500× *g* can’t significantly increase the recover ratio.

Second, for urine sediment microscopy, nephrologists depend on abnormal findings to make a differential diagnosis of renal diseases. In contrast, medical technologists focus on procedural accreditation, i.e., applying the standardized protocol for urine sediment microscopy for an accurate count of urinary RBCs and WBCs. However, reports issued by medical technologists are often unacceptable to nephrologists, because these reports lack a description of dysmorphic RBCs, or they have inaccurate counts of RTE cells and granular casts. This is partly because the recovery ratios for dysmorphic RBCs and RTE cells are insufficient for use by nephrologists, based on medical technologists’ use of an RCF setting of 400× *g* and 12-fold concentration factor. In addition, there is no standardized image of dysmorphic RBCs. This modified protocol, which was based on our observations of urine sediment microscopy, uses the optimal RCF setting (500× *g*) for the recovery of dysmorphic RBCs in cases of haematuria. The increased concentration factor (20-fold) for urine sediment microscopy results in good agreement between the counts by manual observation and those using a flow cytometer.

Next, some studies have suggested the use of a phase contrast microscope to identify dysmorphic RBCS in medical laboratories^15^,^17^. There can be technical and administrative barriers to this approach because of requirements for specific specimen types. The resolution of a phase contrast microscope is lower than that of the bright field microscope; therefore, a phase contrast microscope is unsuitable for evaluating blood smears or parasites and may not be used in some laboratories^28^. In the present study, we compared the performance of bright field and phase contrast microscopy and found no significant difference in the ability to identify dysmorphic RBCs based on these images. Thus, using bright field microscopy alone is acceptable for this purpose if resources are limited. In addition, the images of dysmorphic RBCs provided by this study will be important references for medical technologists to determine the aetiology of haematuria. The ability to identify dysmorphic RBCs can be improved by combining the two approaches (i.e., a bright field microscopy and use of standardized images for RBC classification).

Finally, formed elements are easier to observe with proper staining method under bright field microscope. It can reduce the risk of misclassifying RTE cells as squamous epithelial cells and can increase laboratory technologists’ ability to detect RTE cells and granular casts^1^. Some researchers report that a scoring index for the differential diagnosis of acute tubular necrosis (ATN) resulting from AKI based on the correct counts of RTE cells and granular casts^23^,^29^,^30^. Using Sternheimer stain significantly increased the contrast of formed elements, especially for the RTE cells and granular casts counting, which allowed the different formed elements to be distinguished in urine sediment.

This modified urinalysis protocol can significantly enhance the quality of urinalysis, which has been confirmed by a certified urinary flow cytometer (r ≥ 0.898, P < 0.001). Specifically, the recovery ratio for an accurate cell count of dysmorphic RBCs in clinical specimens from 34.7% to 42.0% (P < 0.001) and the contrast between different formed elements can be increased by the Sternheimer stain using this modified urinalysis protocol. A total of 28 types of isomorphic and dysmorphic RBCs were successfully identified by using a bright field microscope, with results comparable to those using a standard phase contrast microscope. However, this study only focused on type 2 diabetes mellitus patients, our current results will require additional studies using a wider spectrum of urinary tract diseases, for examples, lupus nephritis, polyomavirus nephropathy, and urate nephropathy, toward a guideline or standard for urine sediment microscopy.

## Additional Information

**How to cite this article**: Chu-Su, Y. *et al*. Enhancing the Detection of Dysmorphic Red Blood Cells and Renal Tubular Epithelial Cells with a Modified Urinalysis Protocol. *Sci. Rep.* **7**, 40521; doi: 10.1038/srep40521 (2017).

**Publisher's note:** Springer Nature remains neutral with regard to jurisdictional claims in published maps and institutional affiliations.

## Supplementary Material

Supplementary Table 1

## Figures and Tables

**Table 1:** Different centrifuge forces and their effects on the residual and recovery ratios of dysmorphic RBCs and isomorphic RBCs.

No.	Dysmorphic RBCs				No.	Isomorphic RBCs			
	400× *g*, supernatant residual (%)	500× *g*, supernatant residual (%)	400× *g*, sediment recovery (%)	500× *g*, sediment recovery (%)		400× *g*, supernatant residual (%)	500× *g*, supernatant residual (%)	400× *g*, sediment recovery (%)	500× *g*, sediment recovery (%)
1D	32.8	15.5	52.2	60.9	21I	10.6	16.3	58.3	61.0
2D	18.4	23.8	31.9	34.1	22I	6.3	8.6	36.0	35.2
3D	25.1	36.6	26.0	42.3	23I	8.8	7.0	23.8	27.5
4D	48.3	24.7	26.5	37.8	24I	10.0	4.2	29.5	30.3
5D	62.2	51.7	24.8	29.7	25I	10.9	5.4	51.3	51.9
6D	42.7	32.3	25.8	28.3	26I	31.4	11.2	45.0	62.6
7D	43.5	35.6	48.3	56.0	27I	4.3	1.8	45.5	45.6
8D	66.5	47.5	30.0	37.5	28I	8.7	4.2	53.2	57.6
9D	47.5	41.9	25.1	29.4	29I	3.8	5.8	86.0	87.3
10D	19.9	17.7	35.5	38.7	30I	5.3	2.8	66.1	65.8
11D	8.4	11.2	64.1	66.8	31I	5.7	3.5	84.0	84.4
12D	20.4	18.7	31.4	37.2	32I	15.3	7.9	50.6	52.9
13D	14.7	11.5	57.2	61.3	33I	9.3	0.7	35.0	40.3
14D	27.8	18.0	28.4	35.4	34I	20.4	0.7	59.7	62.6
15D	25.3	19.0	25.5	27.8	35I	16.3	0.5	48.8	55.6
16D	17.3	13.5	41.1	42.0	36I	11.6	0.5	67.7	74.2
17D	23.0	14.4	28.6	38.2	37I	5.8	1.0	35.1	35.2
18D	27.2	14.5	32.9	37.2	38I	8.1	5.3	69.1	71.1
19D	21.4	14.3	27.6	35.3	39I	14.0	7.4	59.0	73.7
20D	23.8	19.0	31.4	63.2	40I	7.9	6.4	69.9	78.7
Mean	30.8 ± 15.8	24.1 ± 12.4	34.7 ± 11.7	42.0 ± 12.5		10.7 ± 6.5	5.1 ± 4.1	53.7 ± 17.1	57.7 ± 17.8
Paired t-test *P* value	0.002*		<0.001*			0.001*		0.002*	

RBCs: red blood cells.

D: dysmorphic RBCs (n = 20); I: isomorphic RBCs (n = 20).

Paired t-test: 95% confidence interval.

Power of test performed with alpha = 0.050.

*P < 0.05.

**Table 2:** Agreement for RBC in HPF counts by Sysmex UF-1000i and manual observation.

Manual	0–4	5–9	10–19	20–29	30–49	50–99	< 100	Total
Sysmex UF-1000i								
0–4	212	7						219
5–9	0	2	3					5
10–19		1	0	4		1		6
20–29			1	0	0			1
30–49			1	1	1	1		4
50–99					2	0	0	1
>100						1	2	3
Total	212	10	5	5	3	3	2	240

RBC counts in a HPF: Sysmex UF-1000i versus manual observation (n = 240).

RBC: red blood cell.

HPF: high-power field.

**Table 3:** Agreement for WBC in HPF counts by Sysmex UF-1000i and manual observation.

Manual	0–4	5–9	10–19	20–29	30–49	50–99	>100	Total
Sysmex UF-1000i								
0–4	221	8	2					231
5–9	0	0	1					1
10–19		0	2	0				2
20–29			0	0	0			0
30–49				2	0	0		2
50–99					0	1	0	1
>100						0	3	3
Total	221	8	5	2	0	1	3	240

WBC counts in a HPF: Sysmex UF-1000i versus manual observation (n = 240).

WBC: white blood cell.

HPF: high-power field.

**Table 4:** Agreement for EC in HPF counts by Sysmex UF-1000i and manual observation.

Manual	0–4	5–9	10–19	20–29	30–49	50–99	>100	Total
Sysmex UF-1000i								
0–4	218	12						230
5–9	0	1	5					6
10–19		0	2	2				4
20–29			0	0	0			0
30–49				0	0	0		0
50–99					0	0	0	0
>100						0	0	0
Total	218	13	7	2	0	0	0	240

EC counts in a HPF: Sysmex UF-1000i versus manual observation (n = 240).

EC: epithelial cell.

HPF: high-power field.

**Table 5:** Characteristics of urine sediment cells with Sternheimer staining.

Type of cell	Colour of the cytoplasm	Colour of the nucleus
Active neutrophil	Colourless	Colourless
Damaged neutrophil	Pale pink	Blue
Squamous epithelial cell	Pale pink	Dense blue
Urothelial cell	Dense purple	Dense purple-blue
Renal tubular epithelial cell	Pink-red	Cyan blue
