# Supplementary material for: Enhancing the Detection of Dysmorphic Red Blood Cells and Renal Tubular Epithelial Cells with a Modified Urinalysis Protocol
Source: Sci Rep. 2017 Jan 11;7:40521. doi: 10.1038/srep40521 (PMC5225455; doi:10.1038/srep40521)
Supplement: Supplementary Table 1 [file srep40521-s1.doc]

**Supplementary Table:**

**Enhancing the Detection of Dysmorphic Red Blood Cells and Renal Tubular Epithelial Cells with a Modified Urinalysis Protocol**

Yu Chu-Su, MS^a,b^, Kenichi Shukuya, MS^c^, Takashi Yokoyama, BS^d^, Wei-Chou Lin, MD^e^, Chih-Kang Chiang, MD, PhD^f,g,**^,

Chii-Wann Lin, PhD^a,*^

^a^ Institute of Biomedical Engineering, National Taiwan University, No. 1, Sec. 4, Roosevelt Rd., Taipei City 10617, Taiwan

^b^ Department of Laboratory Medicine, National Taiwan University Hospital, No. 7, Zhongshan S. Rd., Taipei City 10002, Taiwan

^c^ Department of Clinical Laboratory, The University of Tokyo Hospital, 7-3-1, Hongo, Bunkyo-ku, Tokyo 113-8655, Japan

^d^ Department of Central Clinical Laboratory, Tokyo Women’s Medical University Hospital, 8-1, Kawada-cho, Shinjyuku-ku, Tokyo 162-8666,

Japan

^e^ Department of Pathology and Graduate Institute of Pathology, College of Medicine, National Taiwan University, No. 7, Zhongshan S. Rd.,

Taipei City 10002, Taiwan

^f^ Graduate Institute of Toxicology, College of Medicine, No. 1, Jen-Ai Rd., Taipei City 10002, Taiwan

^g^ Department of Integrated Diagnostics & Therapeutics, National Taiwan University Hospital, No. 7, Zhongshan S. Rd., Taipei City 10002,

Taiwan

| Supplementary Table 1. Different centrifuge forces and their effects on the residual ratio in supernatant and the recovery ratio from sediment for each cell type | | | | | | | | | | | | | | |
| --- | --- | --- | --- | --- | --- | --- | --- | --- | --- | --- | --- | --- | --- | --- |
|  | RBCs | | | |  | WBCs | | | |  | ECs | | | |
| n = 40 | 400 × *g*,  supernatant  residual (%) | 500 × *g*,  supernatant  residual (%) | 400 × *g*,  sediment  recovery (%) | 500 × *g*,  sediment  recovery (%) |  | 400 × *g*,  supernatant  residual (%) | 500 × *g*,  supernatant  residual (%) | 400 × *g*,  sediment  recovery (%) | 500 × *g*,  sediment  recovery (%) |  | 400 × *g*,  supernatant  residual (%) | 500 × *g*,  supernatant  residual (%) | 400 × *g*,  sediment  recovery (%) | 500 × *g*,  sediment  recovery (%) |
| 1D | 32.8 | 15.5 | 52.2 | 60.9 |  | 23.8 | 0.0 | 28.8 | 36.7 |  | 47.5 | 0.0 | 77.5 | 95.0 |
| 2D | 18.4 | 23.8 | 31.9 | 34.1 |  | 0.0 | 0.0 | 25.0 | 30.0 |  | 0.0 | 0.0 | 57.5 | 50.0 |
| 3D | 25.1 | 36.6 | 26.0 | 42.3 |  | 31.7 | 23.8 | 40.0 | 28.8 |  | 0.0 | 47.5 | 57.5 | 47.5 |
| 4D | 48.3 | 24.7 | 26.5 | 37.8 |  | 0.0 | 0.0 | 45.0 | 65.0 |  | 0.0 | 0.0 | 20.0 | 30.0 |
| 5D | 62.2 | 51.7 | 24.8 | 29.7 |  | 31.7 | 39.6 | 42.7 | 67.1 |  | 23.8 | 23.8 | 30.6 | 33.8 |
| 6D | 42.7 | 32.3 | 25.8 | 28.3 |  | 31.7 | 19.0 | 36.7 | 37.5 |  | 9.5 | 10.0 | 10.0 | 59.2 |
| 7D | 43.5 | 35.6 | 48.3 | 56.0 |  | 31.7 | 31.7 | 70.0 | 78.3 |  | 47.5 | 0.0 | 72.5 | 90.0 |
| 8D | 66.5 | 47.5 | 30.0 | 37.5 |  | 47.5 | 23.8 | 50.0 | 53.8 |  | 0.0 | 0.0 | 81.7 | 71.7 |
| 9D | 47.5 | 41.9 | 25.1 | 29.4 |  | 13.0 | 13.0 | 50.0 | 59.5 |  | 5.3 | 0.0 | 38.6 | 40.3 |
| 10D | 19.9 | 17.7 | 35.5 | 38.7 |  | 0.0 | 0.0 | 40.0 | 61.7 |  | 0.0 | 0.0 | 80.0 | 83.8 |
| 11D | 8.4 | 11.2 | 64.1 | 66.8 |  | 0.0 | 47.5 | 60.0 | 50.0 |  | 0.0 | 0.0 | 75.0 | 60.0 |
| 12D | 20.4 | 18.7 | 31.4 | 37.2 |  | 10.6 | 10.6 | 39.4 | 35.0 |  | 0.0 | 0.0 | 72.0 | 71.5 |
| 13D | 14.7 | 11.5 | 57.2 | 61.3 |  | 0.0 | 0.0 | 30.0 | 31.7 |  | 0.0 | 0.0 | 95.0 | 85.0 |
| 14D | 27.8 | 18.0 | 28.4 | 35.4 |  | 27.1 | 0.0 | 31.4 | 34.3 |  | 0.0 | 0.0 | 74.0 | 80.0 |
| 15D | 25.3 | 19.0 | 25.5 | 27.8 |  | 0.0 | 0.0 | 36.0 | 31.0 |  | 0.0 | 0.0 | 82.5 | 87.5 |
| 16D | 17.3 | 13.5 | 41.1 | 42.0 |  | 5.1 | 2.3 | 70.0 | 86.3 |  | 23.8 | 0.0 | 97.5 | 85.0 |
| 17D | 23.0 | 14.4 | 28.6 | 38.2 |  | 0.0 | 0.0 | 38.8 | 53.8 |  | 0.0 | 0.0 | 90.0 | 90.0 |
| 18D | 27.2 | 14.5 | 32.9 | 37.2 |  | 13.8 | 5.6 | 56.9 | 76.9 |  | 17.8 | 5.9 | 62.5 | 73.1 |
| 19D | 21.4 | 14.3 | 27.6 | 35.3 |  | 0.0 | 0.0 | 32.5 | 35.8 |  | 0.0 | 0.0 | 61.7 | 58.3 |
| 20D | 23.8 | 19.0 | 31.4 | 63.2 |  | 5.3 | 1.8 | 71.9 | 77.5 |  | 0.0 | 0.0 | 56.7 | 58.3 |
| 21I | 10.6 | 16.3 | 58.3 | 61.0 |  | 9.0 | 11.1 | 75.6 | 65.4 |  | 14.6 | 11.9 | 25.8 | 18.8 |
| 22I | 6.3 | 8.6 | 36.0 | 35.2 |  | 1.4 | 5.1 | 57.3 | 61.4 |  | 7.3 | 15.8 | 50.4 | 61.3 |
| 23I | 8.8 | 7.0 | 23.8 | 27.5 |  | 47.5 | 13.6 | 35.0 | 27.9 |  | 0.0 | 31.7 | 48.0 | 76.7 |
| 24I | 10.0 | 4.2 | 29.5 | 30.3 |  | 15.8 | 27.1 | 40.0 | 26.4 |  | 9.5 | 0.0 | 60.5 | 55.0 |
| 25I | 10.9 | 5.4 | 51.3 | 51.9 |  | 35.6 | 21.1 | 45.6 | 39.4 |  | 19.0 | 31.7 | 51.0 | 78.3 |
| 26I | 31.4 | 11.2 | 45.0 | 62.6 |  | 73.9 | 19.0 | 71.1 | 77.5 |  | 19.0 | 31.7 | 29.0 | 21.7 |
| 27I | 4.3 | 1.8 | 45.5 | 45.6 |  | 0.0 | 0.0 | 75.8 | 72.9 |  | 0.0 | 0.0 | 16.7 | 18.3 |
| 28I | 8.7 | 4.2 | 53.2 | 57.6 |  | 11.2 | 5.6 | 79.1 | 64.1 |  | 0.0 | 0.0 | 25.0 | 40.0 |
| 29I | 3.8 | 5.8 | 86.0 | 87.3 |  | 11.4 | 19.0 | 91.0 | 96.0 |  | 0.0 | 0.0 | 60.0 | 85.0 |
| 30I | 5.3 | 2.8 | 66.1 | 65.8 |  | 3.7 | 1.2 | 88.0 | 81.8 |  | 47.5 | 0.0 | 62.5 | 65.0 |
| 31I | 5.7 | 3.5 | 84.0 | 84.4 |  | 9.6 | 6.5 | 87.8 | 88.4 |  | 27.1 | 13.6 | 11.8 | 16.8 |
| 32I | 15.3 | 7.9 | 50.6 | 52.9 |  | 13.6 | 13.6 | 83.6 | 95.0 |  | 0.0 | 0.0 | 22.5 | 22.5 |
| 33I | 9.3 | 0.7 | 35.0 | 40.3 |  | 0.0 | 0.0 | 64.1 | 65.2 |  | 0.0 | 0.0 | 70.0 | 60.8 |
| 34I | 20.4 | 0.7 | 59.7 | 62.6 |  | 8.1 | 0.0 | 75.8 | 78.7 |  | 31.7 | 0.0 | 50.0 | 60.0 |
| 35I | 16.3 | 0.5 | 48.8 | 55.6 |  | 13.1 | 0.0 | 50.3 | 54.8 |  | 4.8 | 0.0 | 43.8 | 47.8 |
| 36I | 11.6 | 0.5 | 67.7 | 74.2 |  | 8.2 | 1.6 | 85.6 | 91.2 |  | 0.0 | 0.0 | 15.0 | 13.0 |
| 37I | 5.8 | 1.0 | 35.1 | 35.2 |  | 2.2 | 0.6 | 75.7 | 71.7 |  | 0.0 | 0.0 | 80.0 | 81.0 |
| 38I | 8.1 | 5.3 | 69.1 | 71.1 |  | 3.9 | 2.6 | 78.0 | 81.8 |  | 6.8 | 6.8 | 25.0 | 21.8 |
| 39I | 14.0 | 7.4 | 59.0 | 73.7 |  | 29.4 | 13.6 | 58.6 | 75.2 |  | 4.5 | 4.5 | 33.6 | 35.7 |
| 40I | 7.9 | 6.4 | 69.9 | 78.7 |  | 23.8 | 0.0 | 56.3 | 68.8 |  | 0.0 | 0.0 | 82.5 | 80.0 |
| Mean | 20.8 ± 15.7 | 14.6 ± 13.2 | 44.2 ± 17.4 | 49.8 ± 17.1 |  | 14.9 ± 16.7 | 9.5 ± 12.1 | 56.7 ± 19.6 | 60.4 ± 21.2 |  | 9.2 ± 14.2 | 5.9 ± 11.2 | 53.9 ± 25.1 | 57.7 ± 24.6 |
| Paired t-test  *P* value | < 0.001* | | < 0.001^*^ | |  | 0.032^*^ | | 0.026^*^ | |  | 0.241 | | 0.066 | |

RBCs: red blood cells; WBCs: white blood cells; ECs: epithelial cell; D: dysmorphic RBCs; I: isomorphic RBC

Paired t-test: 95% confidence interval

Power of test performed with alpha = 0.050

^*^ *P* ＜ 0.05
